# Supplementary material for: The Intranigral Infusion of Human-Alpha Synuclein Oligomers Induces a Cognitive Impairment in Rats Associated with Changes in Neuronal Firing and Neuroinflammation in the Anterior Cingulate Cortex
Source: Cells. 2022 Aug 24;11(17):2628. doi: 10.3390/cells11172628 (PMC9454687; doi:10.3390/cells11172628)
Supplement: Supplementary file 1 [file cells-11-02628-s001.zip › cells-1815519-supplementary.pdf]

Supplementary material

Methods

Challenging beam walk test

Rats were tested by the challenging beam walk test in order to assess motor coordination and balance, as previously show (Boi 2020). The testing apparatus consisted of a 2 m wooden beam placed between a starting platform, elevated 40 cm from the floor, and the home cage, with a slope of 15°. Rats were progressively challenged to run across the beam by using three different beam widths, respectively 15, 10 and 5 mm. All rats were trained to run across the different beams for three days before the initiation of the test. During the test day, each performance was videotaped. Briefly, for each beam widths, the rat was placed at the lower end of the beam and the numbers of stepping errors were counted while traversing the beam to reach the home-cage. The total time spent to complete the task and the latency were also evaluated as general indicators of bradykinesia and akinesia. Some animals failed to complete the task within the established time-frame or fell off the beam, reflecting a sensorimotor impairment, as previously reported (refs). In these occurrences, the error score was increased by adding a numerical increment based on the following criteria: (i) 0.25 increment, when the animal completed 75% of the beam; (ii) 0.5 increment, when the animal completed 50% of the beam; (iii) 0.75 when it only completed 25% of the beam.

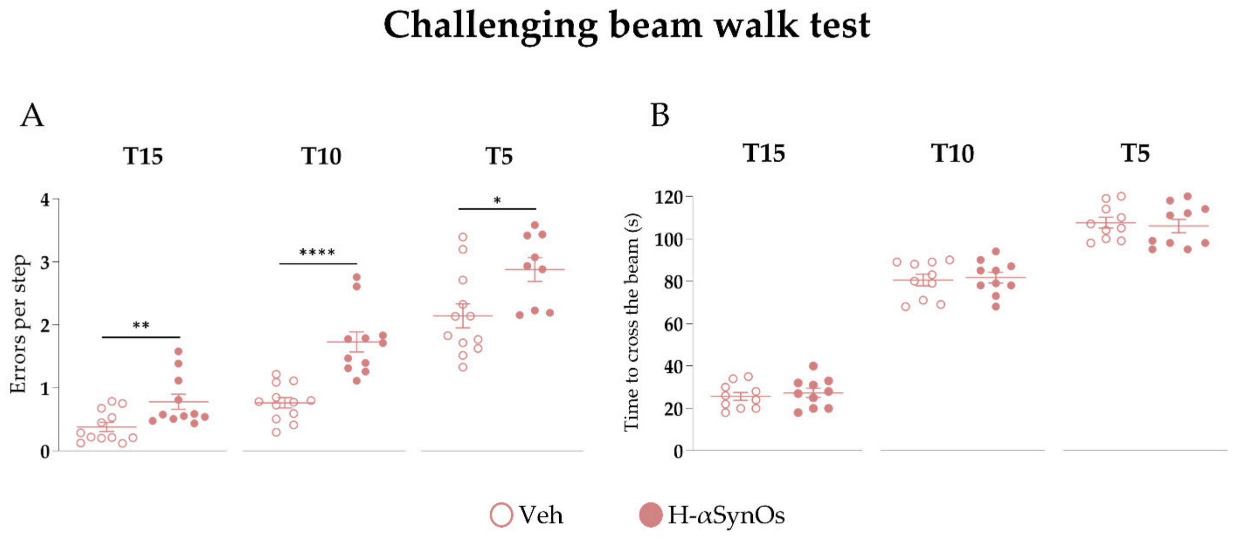

Figure S1. H-αSynOs infused rats developed a significant impairment in motor coordination and balance, but did not show bradykinesia. (A) Error per step made in the three different beam widths. **(B)** Total time spent to cross the beams. No differences were seen among the two experimental groups. Values represent the mean ± SEM. $*p < 0.05$; $**p < 0.01$; $*p < 0.0001$ vs Veh by Unpaired Student's t-test.
